# Engineering Bacteria to Make “Unnatural” Natural Drugs

**DOI:** 10.1371/journal.pbio.0020056

**Published:** 2004-02-17

Faced with new and ongoing threats to public health, researchers are becoming increasingly resourceful in their quest to discover new drugs. Drug researchers have long looked to living organisms for inspiration, either mimicking or extracting chemical formulas from naturally occurring compounds. Bacteria and fungi, for example, produce a wide range of compounds—some of which give them a selective advantage in their own environments—that provide important pharmaceutical activities. One class of these natural compounds are the polyketides, which make up a large portion of the antibiotics (including erythromycin and tetracycline) and antitumor drugs (such as doxorubicin and epothilone) that have been isolated from various microorganisms.

Polyketides are synthesized by bacteria and fungi by the appropriately named polyketide synthases (PKSs). PKSs can be thought of as large molecular factories containing a series of enzymes working on an assembly line: each enzyme in the line adds molecules to a primer, or starter, unit—which is usually an acetate molecule—and then hands off the growing chain to the next enzyme. The specific enzymes set all the characteristics of the polyketide, including the chain length, the building blocks used, and the branching pattern of the molecules. Although microorganisms generate polyketides with a variety of characteristics, one goal of drug discovery research is to increase this diversity even further—a larger pool of polyketides promises more drugs with enhanced pharmaceutical applications.

Early attempts at creating artificial polyketides focused on altering the functional characteristics of naturally occurring polyketides—the length of the chain, the building blocks, and the patterns of the branches. Chaitan Khosla and colleagues have taken this approach one very large step further. Rather than changing the machinery to modify the growing structure of a polyketide, they engineered bacteria to use an alternative, nonacetate primer molecule. This has important practical implications because some medicinally significant compounds do not use the usual acetate primer unit. By dissecting out the specificities of the “starter” and longer, multiunit “elongation” PKS enzymes and by mixing and matching modules, they have produced novel polyketide analogs (in this case, of anthraquinone) with more effective medically relevant properties. One of the compounds they engineered shows enhanced efficacy in blocking the growth of breast cancer cells that depend on the activity of the estrogen receptor, while a second polyketide inhibits an enzyme linked to adult-onset diabetes, demonstrating just two possible new therapeutic applications for synthesized polyketides. But, as the authors propose, this method promises to reveal new pharmaceutical agents that haven't even been discovered yet.

**Figure:**
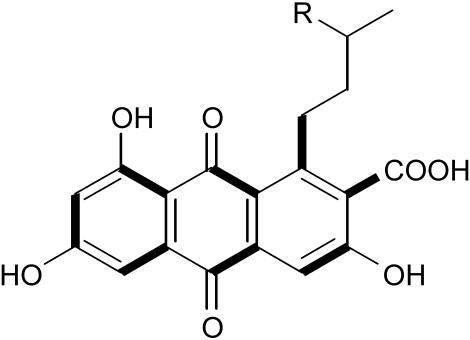
A synthetic polyketide

